# ﻿*Ardisiarecurvipetala* (Primulaceae-Myrsinoideae), a new species from northern Peninsular Malaysia

**DOI:** 10.3897/phytokeys.232.103649

**Published:** 2023-09-13

**Authors:** Avelinah Julius, Mat Yunoh Siti-Munirah, Timothy M. A. Utteridge

**Affiliations:** 1 Institute for Tropical Biology and Conservation, Universiti Malaysia Sabah, Jalan UMS, 88400 Kota Kinabalu, Sabah, Malaysia Universiti Malaysia Sabah Kota Kinabalu Malaysia; 2 Forest Research Institute Malaysia, Kepong, Selangor, 52109, Malaysia Forest Research Institute Malaysia Kepong Malaysia; 3 Herbarium, Royal Botanic Gardens, Kew, Richmond, Surrey, TW9 3AE, UK Royal Botanic Gardens Kew United Kingdom

**Keywords:** Ericales, Malesia, Myrsinaceae, subgenus *Crispardisia*, Taman Negeri Kenyir, taxonomy

## Abstract

Recent fieldwork in Terengganu, Peninsular Malaysia, resulted in the collection of an endemic new species of *Ardisia*, described here as *Ardisiarecurvipetala* Julius, Siti-Munirah & Utteridge. The species is a member of subgenus (§) *Crispardisia* on account of its vascularised glands (bacterial nodules) on the leaf margin and the terminal inflorescence on a specialised lateral branch subtended by a normal leaf (rather than a reduced bract-like leaf). *Ardisiarecurvipetala* is unique amongst all members of §*Crispardisia* by having leaf margins with both vascularised glands and pustule-like structures and can be further distinguished from other Peninsular Malaysian members of this subgenus by the lamina raised between the leaf venation giving a somewhat bullate appearance, unbranched inflorescences, brownish-red pedicels and recurved corolla lobes, each with a creamy-white apex and a small pink patch at the base. *Ardisiarecurvipetala* is known only from a single location in Terengganu and its conservation status is assessed as Data Deficient (DD).

## ﻿Introduction

Botanists at the Forest Research Institute Malaysia (FRIM) are working to produce a more complete and up-to-date Flora for Malaysia, especially by undertaking family revisions for the Flora of Peninsular Malaysia (FPM). To achieve this goal, an ongoing fieldwork programme is being conducted throughout Peninsular Malaysia, especially in areas that are under-explored or have never been botanised, including those that are ecologically significant. This effort has resulted in the discovery of several new species and records for Peninsular Malaysia. For example, four new *Ardisia* Sw. (Primulaceae) species have been discovered since the FPM project launched in 2005 (e.g. Julius and Utteridge (2012, 2021, 2022); Julius et al. (2017)), as well as a new species of *Maesa* Forssk. (Primulaceae; Utteridge (2012)). Other findings include the discovery of the Himalayan genus *Gardneria* Wall. in Pahang and the Neotropical naturalised species *Spigeliaanthelmia* L. (both Loganiaceae) in Selangor (Julius et al. 2013). Many other new species have also been described from various other plant families, including Aristolochiaceae (Yao 2012), Balsaminaceae (Kiew 2016), Capparaceae (Julius 2022), Gesneriaceae (Kiew and Lim 2019; Syahida-Emiza et al. 2020), Orchidaceae (Ong et al. 2020; Ong 2021), Thismiaceae (Siti-Munirah 2021; Siti-Munirah and Dome 2019, 2022; Siti-Munirah et al. 2021) and Zingiberaceae (Sam et al. 2009, 2012; Sam and Ibrahim 2018). This highlights the rich biodiversity of Peninsular Malaysia and opportunities for more discoveries (Middleton et al. 2019).

During a recent botanical survey led by the second author in Taman Negeri Kenyir (Kenyir State Park), Terengganu State, a flowering *Ardisia* plant was documented and collected. The presence of vascularised glands (bacterial nodules) at the incision between the crenatures of the leaf margin and the terminal inflorescence on a specialised lateral branch subtended by a normal leaf (rather than a reduced bract-like leaf) place the taxon within §*Crispardisia*, a well-defined monophyletic group of about 100 species, with a centre of diversity in Asia (Julius et al. 2021; Yang and Hu 2022). After consulting the relevant literature (Larsen and Hu 1991; Hu and Vidal 2004) and specimens of other species in §*Crispardisia*, we confirmed that this is an undescribed species and it is here formally described and illustrated as new to science. It is the only species in §*Crispardisia* that has leaf margins with both vascularised glands and pustule-like structures.

## ﻿Material and method

Morphological description of the new species was based on both fresh and pressed materials; specimens of *Ardisia* have been studied from BKF, BM, K, KEP, L, SAN and SAR. Specimens of related species from SE Asia, especially *A.crispa* (Thunb.) A.DC and species recorded for Peninsular Malaysia. i.e. *A.crenata* Sims, *A.lankawiensis* King & Gamble, *A.minor* King & Gamble, *A.polysticta* Miq, *A.recurvisepala* Julius & Utteridge, *A.ridleyi* King & Gamble, *A.rosea* King & Gamble, *A.sphenobasis* Scheff. and *A.villosa* Roxb. were studied in detail at K and KEP. In addition, specimen images available from JSTOR Global Plants (http://plants.jstor.org/), the Kew Herbarium Catalogue (http://apps.kew.org/herbcat/gotoHomePage.do) and Plants of the World Online (POWO 2023: http://www.plantsoftheworldonline.org/) were examined. Relevant taxonomic literature (e.g. Stone (1982, 1992); Larsen and Hu (1991); Chen and Pipoly (1996); Hu (2002); Hu and Vidal (2004)) was also consulted. The conservation status of the new species was assessed following IUCN Standards and Petitions Committee (2022), including guidelines and procedures developed by FRIM for the Malaysia Plant Red List (Chua and Saw 2006).

## ﻿Taxonomy

### ﻿ Ardisia recurvipetala

Taxon classificationPlantaeEricalesPrimulaceae

Julius, Siti-Munirah & Utteridge, sp. nov. (§ Crispardisia)

10F8D44E-3CFE-595F-B97F-5B4B7A41E235

urn:lsid:ipni.org:names:77326762-1

Figs 1 , 2 , 3

#### Diagnosis.

*Ardisiarecurvipetala* is the only *Ardisia* species with the following combination of characters: leaf margins with both vascularised glands and pustule-like structures, lamina raised between the venation giving a somewhat bullate appearance, inflorescences on the main shoot and specialised lateral branches, white flowers with recurved petals with a pink patch at the base of the corolla lobes.

**Figure 1.**
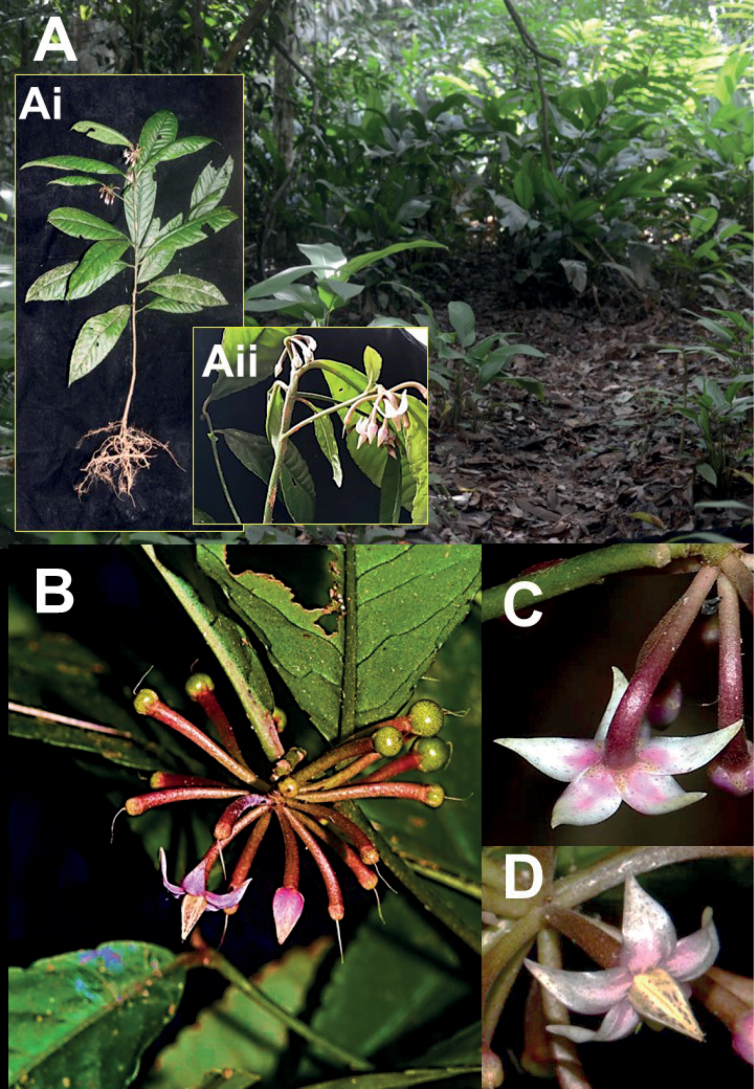
*Ardisiarecurvipetala***A** habitat (inset: **Ai** habit **Aii** inflorescence position on lateral shoot and main stem) **B** lateral branch with flowers and young fruits **C, D** flowers.

#### Type.

Malaysia. Peninsular Malaysia: Terengganu, Hulu Terengganu Distr., Taman Negeri Kenyir, 5°01'00.5"N, 102°32'29.9"E, 201 m elev., 15 June 2022 (fl. & fr.), *Siti Munirah FRI 98670* (holotype KEP!; isotype BORH!).

**Figure 2.**
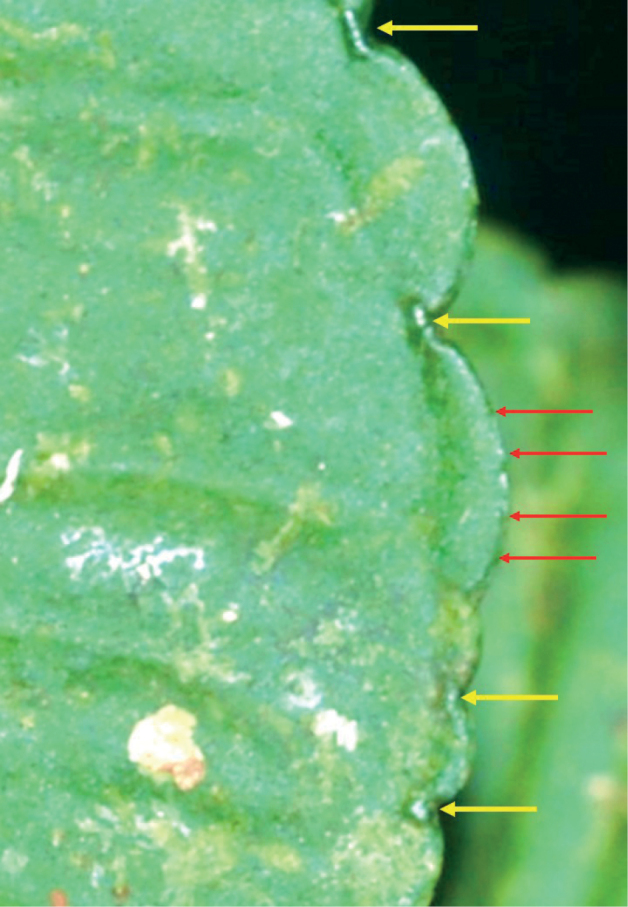
Leaf margin showing vascularised glands (yellow arrow) on the incision between crenation and the pustule-like structures (red arrow) along the crenation.

***Woody shrub*** ca. 1 m tall. ***Branches*** 5–12.5 cm long. ***Indumentum*** absent except for short, white or brown, simple eglandular and glandular hairs on reproductive parts. ***Leaves*** spirally arranged; petioles (0.5–)1–1.5 cm long, winged by the decurrent leaf base, glabrous; lamina chartaceous, with sparse black gland dots throughout abaxially, raised between the venation; usually broadly elliptic, occasionally oblanceolate, (12.5–)17.5–20.5 × 4.5–7.5 cm (excluding reduced leaves subtending inflorescences on specialised lateral branches); young leaves light green, mature leaves dark green above, pale green beneath; base cuneate; margin crenate with vascularised glands (bacterial nodules) at the incision between crenatures and with pustule-like structures along the crenations from projecting venation; apex acute to obtuse; glabrous on both surfaces; mid-rib flat above, raised below; lateral veins 9–14 pairs, irregularly spaced, joining at the marginal vein, distinct on the adaxial surface, prominent on abaxial side; intersecondary veins sometimes present; intercostal veins obscure. ***Inflorescences*** subsessile, terminal on main shoot and on relatively short specialised lateral branches with 1–2(–3) subtending leaves, condensed racemose, ca. 2 × 3 cm, unbranched, 10–18-flowered. ***Flowers*** 5-merous; pedicels brownish-red, 1–1.5 cm long, slender, densely glandular hairy with globular tips, covered with dense, brown gland dots; sepals yellowish-green, pale pink at base, not overlapping, covered with dense brown gland dots, triangular-ovate, 1.5–2 × 1–1.5 mm, sparsely glandular hairy abaxially, glabrous adaxially, margin ciliate with laxly spaced, simple, pale brown hairs, apex obtuse; corolla tube ca. 0.5 mm long, lobes 5, white except the creamy-white apex and pinkish base, covered with dense, brown gland dots, lanceolate, 6–7 × 3–4 mm, glabrous on both surfaces, apex acute, strongly recurved at anthesis; stamens 5, yellowish, subsessile, anthers narrowly lanceolate-mucronate, 5–6 × 1.5 mm, glabrous throughout, gland dotted abaxially, thecae not locellate, dehiscent by longitudinal slits; ovary subglobose, ca. 2 × 1.5 mm, glabrous, ovules ca. 9 arranged in 1-series, style and stigma slender, ca. 5.3 mm long, glabrous. ***Young fruits*** globose, with dense gland dots, 4–6 × 4–6 mm, green, glabrous; pedicels becoming thickened and obconically flared, 1.8–2 cm long. Mature fruits not observed.

**Figure 3.**
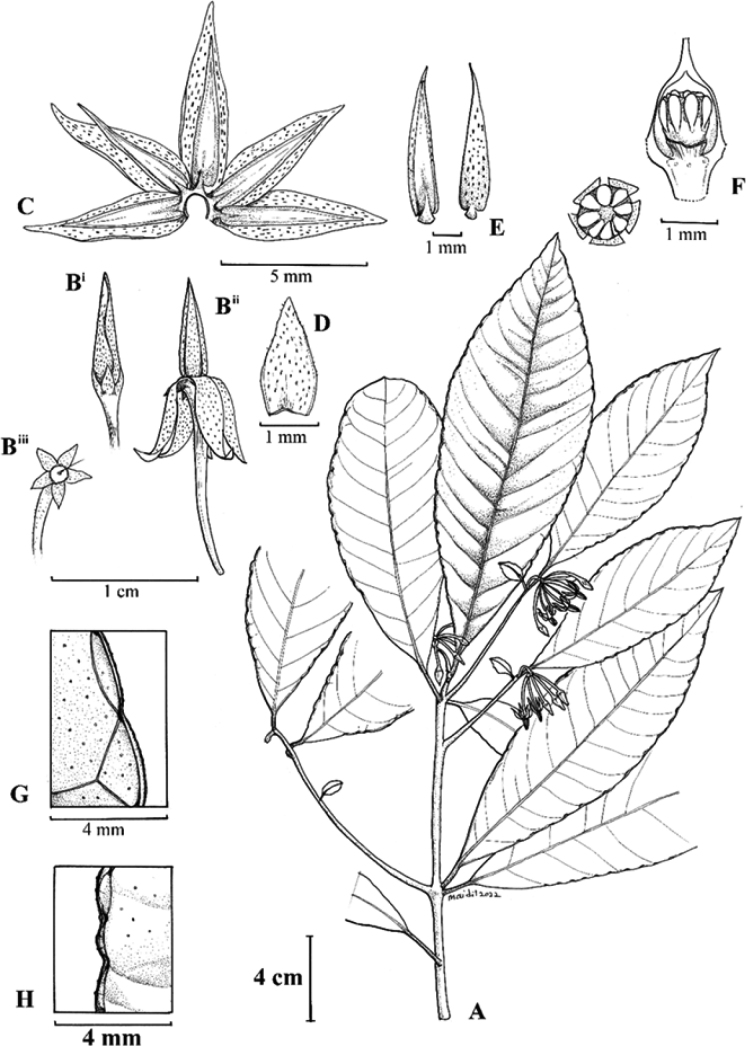
*Ardisiarecurvipetala***A** flowering branches **B** flower bud (**i**), mature flower (**ii**) and flower with corolla removed (**iii**) **C** flower (spread) showing the stamen arrangement **D** adaxial view of sepal **E** ventral (left) and dorsal surfaces (right) of anther **F** anterior (left) and lateral views (right) of ovary **G** leaf margin with venation details and bacterial nodule **H** pustule-like structure on leaf margin crenation. Illustration by Mohd Aidil Nordin.

#### Distribution.

**Figure 4.**
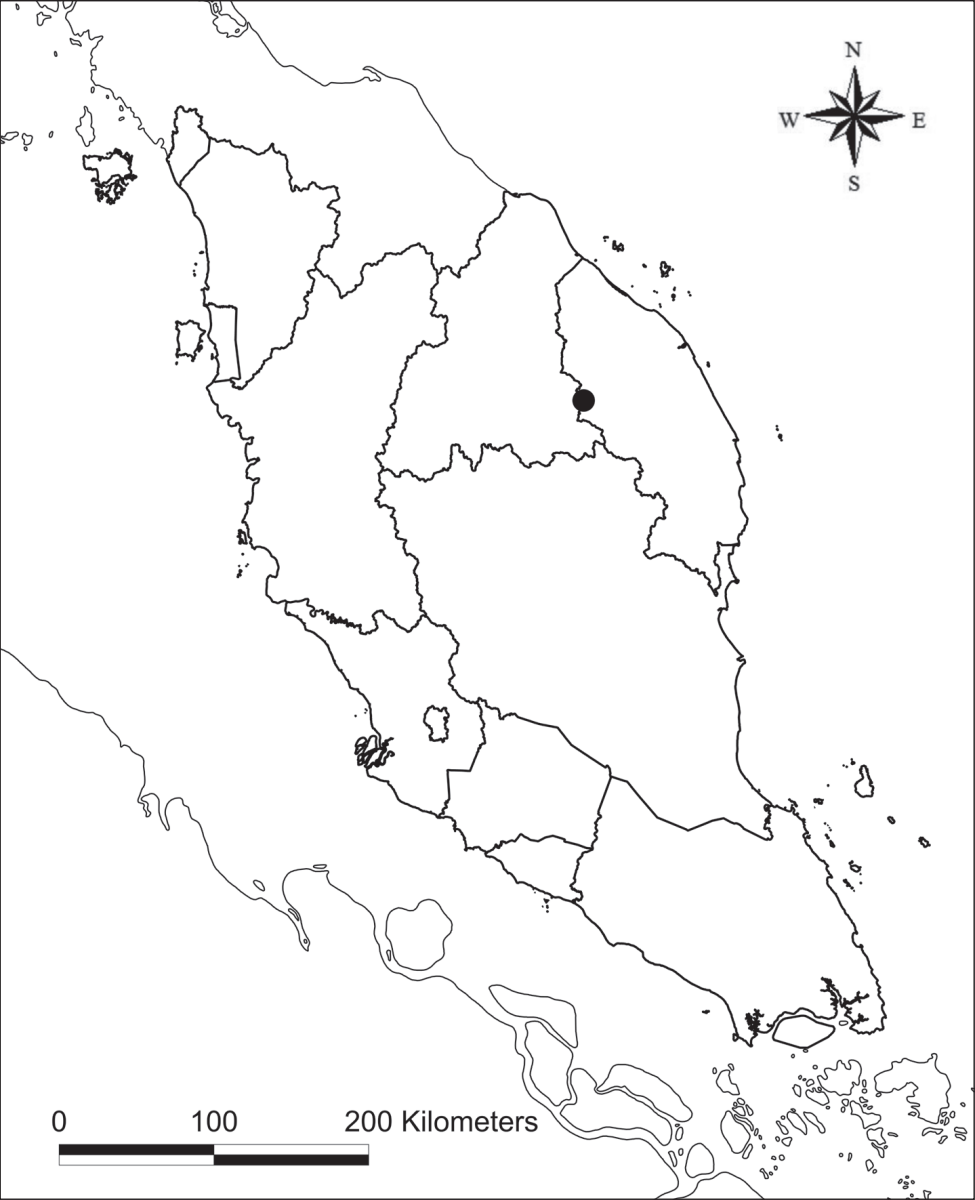
Map showing the type locality of *Ardisiarecurvipetala* in Peninsular Malaysia.

Endemic to Peninsular Malaysia, Terengganu (Taman Negeri Kenyir); currently known only from the type locality (Fig. 4).

#### Ecology.

Growing in a lowland dipterocarp forest at 201 m elev. in a shaded area not far from a small stream.

#### Etymology.

The species epithet is derived from its recurved corolla lobes.

#### Conservation status.

Data Deficient (DD). *Ardisiarecurvipetala* is known from fewer than five individuals in flower and fruit collected from intact forest patches within the Taman Negeri Kenyir. The sites where the taxon was found were previously part of the Tembat Forest Reserve before it was gazetted into Taman Negeri Kenyir in 2018. Further surveys are needed to understand the threats at the type locality and if the species is distributed outside the current area and, until these data are obtained, the species is assessed as Data Deficient (DD) (Chua and Saw 2006; IUCN Standards and Petitions Committee 2022).

#### Notes.

Peninsular Malaysian members of §*Crispardisia* can be placed into two informal groups, based on inflorescence position (Stone 1989; Julius and Utteridge 2021). The first group has inflorescences strictly terminal on specialised lateral leafy branches, while the second group has inflorescences lateral (axillary) and/or terminal on the main stem and/or lateral branches. *Ardisiarecurvipetala* falls into the second group, in which the majority of species in §*Crispardisia* belong, including six species from Peninsular Malaysia. Other than characters mentioned in the diagnosis, the new species can be easily recognised by its unbranched and subsessile inflorescences terminal on the main shoot and on relatively short specialised lateral branches with 1 to 2(–3) subtending leaves, tiny sepals (1.5–2 × 1–1.5 mm) that are yellowish-green and pinkish at base and corolla lobes that are strongly recurved at anthesis (Figs 1B, 3Bii).

Unlike other members of §*Crispardisia*, the leaf margins of the new species have both vascularised glands at the incisions between the crenatures and pustule-like structures along the crenations (Fig. 2); these result from the venation projecting from the leaf margin and should not be mistaken for bacterial nodules. This combination of vascularised glands and pustule-like structures is not observed in other Peninsular Malaysian members of §*Crispardisia*.

The new species is similar to *Ardisiasphenobasis* in having 17.5–23 cm long leaves (excluding reduced leaves subtending inflorescences on specialised lateral branches), but it has inflorescences that are terminal on the main shoot and specialised lateral branches (vs. strictly lateral on main stem). The whitish corollas of the new species are somewhat similar to those of *A.villosa* and *A.crenata*. However, *A.recurvipetala* (Fig. 3) differs in its largely glabrous flowers, except for short, simple eglandular and glandular hairs on the pedicels and calyx (vs. hairy throughout with long, villous hairs in *A.villosa*). Compared to *A.crenata*, a widespread species with a whitish to pinkish corolla, branched and unbranched inflorescences and a flat and coriaceous lamina, the new species has corolla lobes with a creamy-white apex and a pink patch at the base, strictly unbranched inflorescences and chartaceous leaves with the lamina raised between the venation.

This latest addition brings the number of §*Crispardisia* species native to Peninsular Malaysia to ten. Of these, four species, including the new one, are endemic to Peninsular Malaysia: *A.lankawiensis* King & Gamble, *A.minor* King & Gamble, *A.recurvipetala* and *A.recurvisepala* Julius & Utteridge.

We consider species of *Ardisia* as important indicators of tropical and subtropical forest quality. Whilst some species are found in a range of habitats (e.g. *A.elliptica* Thunb.), many species have restricted distributions and habitat requirements, including several species of §*Crispardisia*. For example, in Peninsular Malaysia, *A.lankawiensis* is restricted to limestone habitats on Langkawi Islands and, in Thailand, *A.pilosa* H.R.Fletcher is restricted to the subtropical forests found on only Phu Kradeung mountain in north-east Thailand. That this new taxon is currently only found in unlogged areas within the Taman Negeri Kenyir suggests that *A.recurvipetala* is a useful indicator of primary forest within the forest reserve boundary. The discovery and description of new species is important. It contributes to naming and documenting our local biodiversity as part of a revision of the Primulaceae for the FPM, provides a better understanding of botanical distribution patterns and results in information about forest quality in Peninsular Malaysia.

## Supplementary Material

XML Treatment for Ardisia recurvipetala

## ﻿Additional information

### Author ORCIDs

Avelinah Julius https://orcid.org/0000-0003-1991-1915

Siti-Munirah Mat Yunoh https://orcid.org/0000-0002-5062-9988

Timothy M. A. Utteridge https://orcid.org/0000-0003-2823-0337

### Data availability

All of the data that support the findings of this study are available in the main text.
